# Vancomycin resistant *Streptococcus equi* subsp*. equi* isolated from equines suffering from respiratory manifestation in Egypt

**DOI:** 10.14202/vetworld.2021.1808-1814

**Published:** 2021-07-14

**Authors:** Amany A. Arafa, Riham H. Hedia, Nagwa S. Ata, Eman S. Ibrahim

**Affiliations:** Department of Microbiology and Immunology, National Research Centre, Dokki, Egypt

**Keywords:** antibiotic resistance, equines, polymerase chain reaction, *Streptococcus equi* subsp*. Equi*, vancomycin

## Abstract

**Background and Aim:**

Upper respiratory tract infections are common in horses and can be caused by a variety of pathogens, mainly *Streptococcus equi* subsp*. equi*, which are a significant equine pathogen causing major health issues as well as financial losses to the equine industry. This study aimed to determine the prevalence of *Streptococcal* bacteria in equines in Egypt, and characterize vancomycin-resistant *S. equi* subsp*. equi* phenotypically and genotypically.

**Materials and Methods:**

*S. equi* subsp*. equi* was isolated from internal nares of horses. All strains were confirmed by polymerase chain reaction-based detection of *Streptococcus* genus-specific *16S rRNA*, *sod*A and *see*I genes. Antibiotic susceptibility was determined phenotypically using the disk diffusion method. Genotypic detection of antibiotic resistance genes was performed by analyzing as b-lactamase resistance (*bla*Z), tetracycline resistance (*tet*K), vancomycin resistance (*van*A), and chloramphenicol resistance (*fex*A).

**Results:**

Eight streptococcal isolates were confirmed as *S*. *equi* subsp*. equi*. The genotypic characterization of antibiotic resistance showed resistance to *van*A and *tet*K, with a frequency of 87.5% and 12.5%, respectively, while the frequency of sensitivity was 100% for *blaz* gene and *fex*A gene.

**Conclusion:**

In this study, we assessed vancomycin-resistant *S. equi* subsp*. equi* from equines suffering from respiratory manifestation in Egypt.

## Introduction

Upper respiratory tract infections are common in horses and can be caused by a variety of pathogens, including viruses, fungi, and bacteria. *Streptococcus equi* is a significant equine pathogen known as streptococci of Lancefield Group C with two subspecies with major clinical importance in horses: *S. equi* subsp*. equi* and *S. equi* subsp*. zooepidemicus*. The *S. equi* has four prophage-encoded superantigens/toxins, *See*H, *See*I, *See*L, and *See*M and the latter three stimulate the proliferation of equine peripheral blood mononucleated cells *in vitro*, triggering an immune response [1]. Strangles is a highly infectious and serious nasopharyngeal disease in horses caused by *S. equi* subsp*. equi*. It is the most commonly diagnosed infectious disease in horses worldwide, causing major health issues as well as financial losses to the equine industry [2,3]. Inhalation or direct contact with mucopurulent discharge from an infected animal transmits the disease resulting in fever, depression, and swelling of the submandibular and retropharyngeal lymph nodes, potentially contributing to respiratory distress [4]. Complications include secondary cellulitis at external abscessation sites, empyema of the guttural pouch and its carrier state persistence, metastatic abscessation, purpura hemorrhagica, emergency tracheostomies, and unusually secondary *S. equi* pneumonia or myositis [4]. Streptococci can be host-specific or spread between many species, including zoonotic transmission to humans causing disease in them. The organism may be carried on clothes, boots, or unwashed hands by a person handling an infected horse. In the case of human beings, *S. equi* subsp*. equi* causes invasive infections in immunocompromised hosts, often after direct contact with horses. Such outbreaks are linked to increased mortality and poor neurological outcomes among survivors. Beta-lactam antimicrobial agents are the foundation for treatment, while neurosurgical interference is sometimes needed [5]. One of the most critical problems facing equine practitioners is effective control that helps prevention of highly contagious *S. equi* subspecies *equi* infections or strangles. To prevent the rapid spread and complications associated with the disease, the rate of clinical and differential diagnosis is very important because most of the respiratory tract diseases in equines are contagious. Unfortunately, considering the high population of equines and their value, very few studies have been carried out in Egypt, especially on methods for rapid clinical and differential diagnosis of respiratory tract infections [6].

The use of polymerase chain reaction (PCR), a method for detecting small amounts of DNA, provides us with a very quick and useful way to determine *S. equi* by amplifying a specific *S. equi* gene [7]. PCR test is a confirmatory test for the identification of *S. equi* subsp*. equi*, the causative agent of strangles, in addition to clinical and bacteriological examination [8]. Penicillin is known to be the drug of choice for the treatment of strangles [9]. Besides that, ceftriaxone, ceftiofur, cefquinome, and cefotaxime showed high *in vitro* efficacy against *S. equi* subsp*. equi* [8,10]. There have been no studies that provide feedback on the use of vancomycin for the treatment of strangles. In sensitive bacteria, vancomycin suppresses the second stage of cell wall formation [11]. Furthermore, there is evidence that vancomycin affects cell membrane permeability and inhibits ribonucleic acid synthesis selectively [11]. *Streptococcus pneumoniae* (including multiple resistant variants), *Streptococcus pyogenes*, *Streptococcus* *agalactiae*, and *Streptococcus bovis* are all susceptible to vancomycin [11].

The present study aimed to determine the frequency of *Streptococcal* bacteria in equines in Egypt, and characterize *S. equi* subsp*. equi* with respect to their antibiotic resistance profile phenotypically and genotypically.

## Materials and Methods

### Ethical approval

Ethical approval was not required for this study; however, samples were collected as per standard sample collection procedure.

### Study period and location

Nasal swabs were collected from February 2019 to January 2020 from horses with respiratory manifestation from the equine farm in El Haram, Giza,, Egypt. The samples were processed at the Department of Microbiology and Immunology, National Research Centre.

### Samples

A total number of 159 nasal swabs classified into three groups: foreign breed (29), native breed (73), and Arabic breed (57) were used. Nasal swabs were collected using sterile cotton swabs moistened with normal saline from the back of the horse’s nasal cavity (nasopharynx). The samples were carefully wrapped, numbered, and sent to the laboratory as quickly as possible in an icebox.

### Isolation and phenotypic identification of *S. equi* subsp. *equi*

Nasal swabs were inserted into brain heart infusion broth, incubated overnight at 37°C and then cultured on blood agar and Staph Strept media at 37°C for 24 h. The colonies’ morphological characteristics, appearance, and hemolytic activity were all studied. Smears of suspected colonies were prepared and stained with Gram’s stain for microscopic examination before being transferred to semisolid slope agar for biochemical identification using catalase test, oxidase test, and Lancefield grouping [12].

### Antimicrobial assay

The isolates were checked for susceptibility to six antimicrobial agents that are widely used to treat equines in Egypt and are considered by the WHO to be “the most effective medicines” still in use in healthcare settings [13]. This included: Penicillin (10 U); ampicillin (10 μg); tetracycline (30 μg); chloramphenicol (30 μg); sulfamethoxazole-trimethoprim (23.75–1.25 μg); and vancomycin (30 μg). The disk diffusion method on Mueller–Hinton agar (Oxoid) was used, according to the Clinical Laboratory Standards Institute 2020 [14].

### Genotypic characterization

#### Molecular confirmation of S. equi subsp. equi identity

All *Streptococcus* isolates were subcultured on blood agar plate and incubated at 37°C for 16-18 h. DNA was extracted from isolates using the QIAamp DNA Mini kit (Qiagen, Germany, GmbH) with some modifications to the manufacturer’s recommendations. At 56°C for 10 min, 200 μL of the sample suspension was incubated with 10 μL of proteinase K and 200 μL of lysis buffer. After incubation, the lysate was mixed with 200 μL of 100% ethanol. Following the manufacturer’s instructions, the sample was washed and centrifuged. The nucleic acid was eluted with 100 μL of the kit’s elution buffer. Three primer pairs were used in multiplex PCR, one of which targeted the *Streptococcus* genus-specific *16S rRNA* gene (fragment of 912 bp), according to Osakabe *et al*. [15]. The second pair of primers targeted the superoxide dismutase A encoding gene *sod*A for the identification of both subspecies *S. equi* subsp*. zooepidemicus* and *S. equi* subsp*. equi* (235 bp fragment). The third primer targeted the gene *see*I encoding the exotoxins *See*I which is a marker for *S. equi* subsp*. equi* but not *S. equi* subsp*. Zooepidemicus* (fragment of 520bp; [16]). Each PCR tube was prepared in 50 μL reaction containing 25 μL of EmeraldAmp Max PCR Master Mix (Takara, Japan), 1 μL of each primer at 20 pmol concentration, 14 μL of water, and 5 μL of DNA template. An Applied Biosystems 2720 thermal cycler was used to conduct the reaction. PCR reaction condition is mentioned in Table-1 [15-19].

**Table-1:** Primers and PCR reaction conditions used for the genotypic analysis of streptococcal isolates.

Target gene	Primers sequences	PCR product size (bp)	Primary Denaturation	Amplification (35 cycles)			Final extension	Reference

				Secondary denaturation	Annealing	Extension		
Streptococcus *16S rRNA*	CGGGGGATAACTATTGGAAACGATA	912	94°C	94°C	57°C	72°C	72°C	[15]
	ACCTGTCACCCGATGTACCGAAGTA		5 min	30 s	40 s	50 s	10 min	
*sod*A	CAG CAT TCC TGC TGA CAT TCG TCAGG	235						[16]
	CTG ACC AGC CTT ATT CAC AAC CAG CC							
*see*I	GAA GGT CCG CCA TTT TCA GGT AGT TTG	520						
	GCA TAC TCT CTC TGT CAC CAT GTC CTG						
*tet*K	AGCTGCATTTCCAGCACTCG	352	94°C	94°C	55°C	72°C	72°C	Designed in this study
	CAGGAATGACAGCACGCTAAC		5 min	30 s	40 s	40 s	10 min	
*van*A	CATGACGTATCGGTAAAATC	885	94°C	94°C	50°C	72°C	72°C	[18]
	ACCGGGCAGRGTATTGAC		30 s	40 s	50 s	10 min	
*bla*Z	TACAACTGTAATATCGGAGGG	833	94°C	94°C	50°C	72°C	72°C	
	CATTACACTCTTGGCGGTTTC		5 min	30 s	40 s	50 s.	10 min	[17]
*fex*A	GTA CTT GTA GGT GCA ATT ACG GCT GA	1272	94°C	94°C	56°C	72°C	72°C	[19]
	CGC ATC TGA GTA GGA CAT AGC GTC		5 min	30 s	40 s	1.2 min	12 min	

#### Molecular characterization of antibiotic-resistant genes

Eight *S. equi* subsp*. equi* isolates were examined to detect the presence of genes associated with the monitored antibiotic resistances: The b-lactamase *bla*Z gene (PEN resistance) was determined by primers designed according to Bagcigil *et al*. [17]; *tet*K (TET resistance) that confer resistance to tetracycline was determined by primers designed according to the GenBank accession number CP031556 using Primer Quest® design tool; *van*A that confer resistance to ­vancomycin was determined by primers designed according to Patel *et al*. [18]; and *fex*A gene that confers resistance to chloramphenicol was determined by primers designed according to Kehrenberg and Schwarz [19]. Each antibiotic resistance gene was examined by uniplex PCR. Primers were utilized in a 25-μL reaction tube containing 12.5 μL of EmeraldAmp Max PCR Master Mix (Takara, Japan), 5.5 μL of water, 1 μL of each primer of 20 pmol concentrations, and 5 μL of DNA template. Reactions for amplification of each resistance gene are shown in Table-1. After electrophoresis on a 2% agarose gel containing 0.5 g/mL ethidium bromide, the PCR amplicons were simultaneously visualized and resolved using an ultraviolet lightbox, and the reagent control was used in all PCR assay runs.

## Results

### Phenotypic results

A small translucent colony, some of which may be mucoid, with Gram’s stain was detected as Gram-positive cocci occurring in pairs and short chains when examined under a light microscope. As shown in Table-2, the result of isolation of Gram-positive bacteria was 55 isolates (19 isolates from foreign breed, 26 isolates from native breed, and ten isolates from Arabic breed).

**Table-2:** Prevalence of Gram-positive bacteria recovered from nasal swabs from foreign, native, and Arabic horse breeds.

Type of horses	Gram-positive bacteria	%
Foreign breed (29)	19	65.5
Native breed (73)	26	35.6
Arabic breed (57)	10	17.5

#### Biochemical testing results

The results of biochemical identification and the biochemical characteristics are shown in Table-3. There were three suspected isolates of *S. equi* subsp. *equi* recovered from a foreign breed with a frequency of 15.7%, and five suspected isolates of *S. equi* subsp. *equi* recovered from a native breed with a frequency of 19.23%.

**Table-3:** Biochemical identification of the suspected 55 Gram-positive bacteria recovered from nasal swabs from foreign, native, and Arabic horse breeds.

Test	Isolates from Foreign breed (19)		Isolates from Native breed (26)		Isolates from Arabic breed (10)	
		
	No. of positive isolates	%	No. of positive isolates	%	No. of positive isolates	%
Catalase	0	0	0	0	0	0
Hemolysis	3	15.7	5	19.23	0	0
Oxidase	0	0	0	0	0	0
Lancefield group c	3	15.7	5	19.23	0	0

#### Antimicrobial resistance results

The results observed are shown in Table-4. The frequency of antibiotic resistance against vancomycin was the highest at 87.5%, while the frequency of the susceptibility against ampicillin and Sulfamethoxazole –trimethoprim was at 100%. There was an intermediate resistance observed against Chloramphenicol, Tetracycline, and penicillin at 12.5%, 25%, and 50%, respectively.

**Table-4:** Phenotypic and genotypic pattern of antibiotic resistance of *Streptococcus equi* subsp. *equi*.

*Streptococcus equi* subsp. *equi* (Isolates)	Phenotypically (Disk diffusion test)						Genotypically (PCR)			
	
	P	AMP	T	V	C	SXT	*bla*Z	*tet*K	*van*A	*fex*A
1	S	S	S	R	S	S	−	−	+	−
2	S	S	S	R	S	S	−	−	+	−
3	I	S	S	R	S	S	−	−	+	−
4	I	S	S	S	S	S	−	−	+	−
5	S	S	S	R	I	S	−	−	−	−
6	I	S	I	R	S	S	−	−	+	−
7	I	S	I	R	S	S	−	+	+	−
8	S	S	S	R	S	S	−	−	+	−

P=Penicillin (10 U), AMP=Ampicillin (10 μg), T=Tetracycline (30 μg),V=Vancomycin (30 μg), C=Chloramphenicol (30 μg), SXT=Sulfamethoxazole -trimethoprim (23.75-1.25 μg)

### Genotypic Results of multiplex PCR for confirming *S. equi* subsp. *equi*

**Figure-1:**
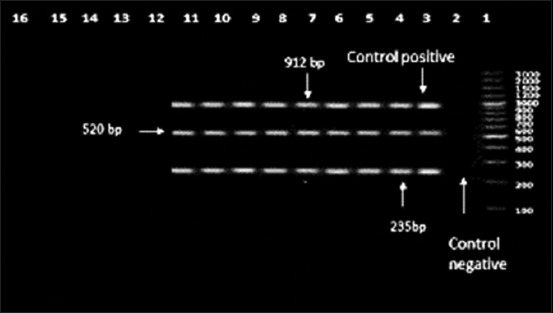
Agarose gel electrophoresis using multiplex PCR. Showing amplification of 912 bp, 235 bp and 520 bp fragments for *Streptococcus* *16S rRNA*, *sod*A and *see*I genes respectively carried out with their specific primer. Lane 1:100 bp DNA ladder. Lane 2: Control Negative [*Escherichia coli* DNA (NCIMB 50034)]. Lane3: Control Positive (*S. equi subsp. equi*) shows Positive amplification of both *16S rRNA*, *sod*A and *see*I genes at 912 bp, 235 bp, and 520 bp fragments from the extracted DNA of *S. equi* subsp. *equi*. Lanes 3-11: shows positive amplification of 912 bp, 235 bp and 520 bp fragment for *16S rRNA*, *sod*A and *see*I genes.

Eight *S. equi* subsp*. equi* strains were confirmed by PCR amplification of Streptococcus *16S rRNA* gene (the PCR fragment length was 912bp), *sod*A and *see*I genes (the PCR fragment length was 235bp and 520bp, respectively) as shown in Figure-1.

### Results of genotypic antimicrobial resistance patterns

**Figure-2:**
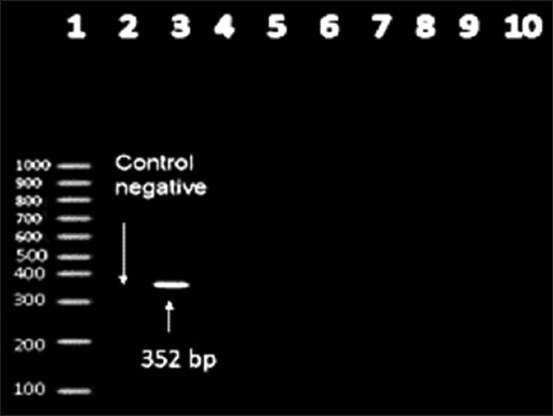
Agarose gel electrophoresis showing amplification of 352 bp fragment for *tet*K gene performed with its specific primer. Lane 1:100 bp DNA ladder. Lane 2: Control Negative [*Escherichia coli* DNA (NCIMB 50034)]. Lanes 3: Positive amplification of 352 bp fragment for tet K gene. Lanes 4-10: Negative amplification of 352 bp fragment for tet K gene.

**Figure-3:**
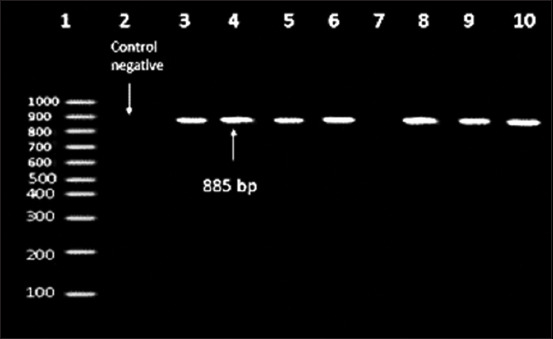
Agarose gel electrophoresis showing amplification of 885 bp fragment for *van*A gene performed with its specific primer. Lane 1:100 bp DNA ladder. Lane 2: Control Negative [*Escherichia coli* DNA (NCIMB 50034)]. Lanes 3-6, 8-10: Positive amplification of 885 bp fragment for *van*A gene. Lane 7: Negative amplification of 885 bp fragment for *van*A gene.

Screening the all *S. equi* subsp*. equi strains to van*A gene (the PCR fragment length was 885 bp), resulting in 87.5% positivity while *tet*K gene (the PCR fragment length was 352 bp) was 12.5% positivity but they were negative to *blaz* gene and *fex*A as observed in Figures-2 and 3.

## Discussion

Strangles (equine distemper) is a highly infectious upper respiratory tract disease caused by *S. equi* subsp*. equi* [12]. Strangles can affect horses of any age, although it is most prevalent in weanling foals and yearlings, who have more severe clinical signs. For the treatment of strangles, penicillins have long been regarded as the medicine of choice [9]. Antimicrobial resistance develops as a natural result of antimicrobial use in a variety of industries, including human health, animal health and production, aquaculture, and agriculture [20]. Because of the rapid emergence and dissemination of resistant bacteria and associated antibiotic-resistant genes among humans, animals, and the environment, antibiotic resistance is considered as a critical multifactorial and dynamic global issue [21]. As a result, we set out to determine the prevalence of Streptococcal bacteria in Egyptian horses, and characterize *S. equi* subsp*. equi* in terms of their antibiotic resistance profile, both phenotypically and genotypically.

In the present study, the frequency of *S. equi* was 8/55 (14.5%), while there was a high prevalence of *S*. *equi* from nasal swabs; 22/57 (39%) by Lindahl [22], 28/48 (58%) by Delph *et al*. [23], and low prevalence of *S. equi* subsp. *equi*, 2/40 (5%) by Javed *et al*. [24]. In a study by Erol *et al*. [25], a total of 2497 β-hemolytic streptococci were isolated from 2391 cases, *S. equi* subsp*. equi* (5.8%). Also, in Egypt, a recent study in 2019 [26] reported five suspected isolates of *S. equi* subsp*. equi* recovered from a foreign breed with a prevalence of 17.3%, and 19 suspected isolates of *S. equi* subsp*. equi* recovered from a native breed with a prevalence of 61.3%. Our study recorded three isolates of *S. equi* subsp*. equi* recovered from a foreign breed with a prevalence of 15.7% and five isolates of *S. equi* subsp*. equi* recovered from a native breed with a prevalence of 19.23%. Mohamed *et al*. [6] recorded that the frequency of *S. equi* subsp*. equi* and *S. equi* subsp. *Zooepidemicus* infections among the total animal population was 11.83% and 4.96%, respectively. PCR technique showed high sensitivity and specificity for the detection of *S. equi* species. The difference in frequency rate of *S. equi* subsp*. equi* may be due to age, season [6] or the difference in biosecurity measures, control of outbreaks and fast diagnosis using recent techniques such as PCR [9].

In our study, as shown in Table-4, the antibiotic sensitivity tests showed that the frequency of antibiotic resistance against vancomycin was the highest at 87.5% which indicates the development of vancomycin resistance. On the other hand, Nasr and Arafa [27] in their study have mentioned that the isolated Egyptian isolates of *S. equi* subsp*. equi* showed susceptibility to vancomycin, erythromycin, clindamycin, and chloramphenicol. In our study, there was an intermediate resistance against chloramphenicol of about 12.5%.

In addition, our study revealed that the frequency of the susceptibility against ampicillin and sulfamethoxazole-trimethoprim was 100%. There was an intermediate resistance against tetracycline and penicillin at 25% and 50%, respectively, while Erol *et al*. [25] reported that *Streptococci* were found to be generally susceptible to cephalothin, erythromycin, nitrofurantoin, penicillin, ticarcillin, and clavulanate, according to the Kirby–Bauer disk diffusion susceptibility test protocol.

A study in 2016 by Javed *et al*. [24] showed the antibiotic resistance of two isolates of *S. equi* subsp*. equi* against penicillin G; all other isolates were found susceptible to streptomycin and amoxicillin. Recently, Fonseca *et al*. [28] detected resistance to penicillin as well as tetracycline among *S. equi* subsp*. equi* from the upper respiratory tract samples at a percentage of 12.5% and 62.7%, respectively. On the other hand, *S. equi* subsp*. zooepidemicus* and *S. equi* subsp*. equi* were susceptible to ceftiofur and erythromycin at a percentage of 100% and 99%, respectively. Furthermore, Yaghoobpour *et al*. [29] reported that the highest rate of resistance in both *S. equi* subsp*. equi* and *S. equi* subsp*. zooepidemicus* were observed against amoxicillin, while the highest rate of sensitivity was to ceftriaxone.

In Egypt, a recent study in 2019 [26] showed that the recovered *S. equi* and *S. zooepidemicus* isolates were sensitive to cefoxitin, gentamicin, and ciprofloxacin, while they show high resistance to vancomycin, which agreed with the results recorded in the present study, wherein the resistance against vancomycin was 87.5% and this was similar to Seady *et al*. [30], who reported high resistance to vancomycin (70%).

In our study, all the eight streptococcal isolates were subjected to genus-specific *16Sr RNA* PCR for confirmation as streptococci and the PCR fragment was at 912 bp. New methods for bacterial identification have emerged due to recent advances in nucleic acid technology, such as PCR and *16S rRNA* analysis. Numerous publications had previously employed PCR-mediated identification based on ­species-specific regions of the *16S rRNA* gene to identify several streptococcal species [31-34]. Sequencing of internal portions of the *16S rRNA* gene from *S. equi* subsp*. zooepidemicus* and *S. equi* subsp*. equi* indicated that certain strains of both subspecies have identical or nearly identical *16S rRNA* gene sequences, and that certain *S. equi* subsp*. zooepidemicus* displayed intraspecies variation in this area [35] showed that this target gene could not or could only be utilized inadequately for subspecies identification and differentiation. Furthermore, the 16S–23S rDNA intergenic spacer region, a second target for PCR-mediated identification, appears to be very heterogeneous [36,37] for *S. equi* subsp*. zooepidemicus*, preventing the construction of species-specific oligonucleotide primers. For *S. equi subsp. equi*, a PCR-based identification based on the M-like protein gene has been published [38,39].

Further, in our study, all eight streptococcal isolates were positive for *sod*A encoding gene at 230bp and *see*I gene at 520bp, and this agreed with results of Alber *et al*. [40]. The multiplex PCR presented here should help to boost the *S. equi* subspecies recognition in future infections in animals and humans.

In our study, the genotypic antimicrobial resistance patterns agreed with the phenotypic antibiotic-resistant pattern, wherein screening of the *S. equi* subsp*. equi* strains for *van*A gene (the PCR fragment length was 885 bp) resulted in 87.5% positivity, while that for *tet*K gene (the PCR fragment length was 352 bp) was 12.5% positivity, but was negative for *bla*Z and *fex*A genes.

## Conclusion

*Streptococcus equi* subsp*. equi* is an important pathogen affecting equines and can easily be transmitted, causing outbreaks. It also has zoonotic importance, and therefore a quick diagnosis of the infected cases using PCR is necessary to isolate positive cases and avoid transmission to other healthy ones. In Egypt, we detected antibiotic resistance strains of *S. equi* subsp*. equi*, the most critical vancomycin resistance strains, which give us an alarm of the danger we face from the strains of bacteria that are difficult to be controlled by the use of antibiotics. In the medical field, antibiotics are overprescribed all over the world, despite concerns of overuse, so we recommend antibiotic sensitivity test before starting treatment. Moreover, we should apply more research to study the vancomycin-resistant strains as it is very dangerous not only to animal health but also to human health worldwide.

## Authors’ Contributions

NSA: Designed and revised the manuscript critically. ESI: Collected samples and then performed the bacterial isolation and biochemical typing. RHH: Antibiogram assay, DND extraction, PCR, drafted, revised, and finalized the manuscript for submission. AAA: Designed the study, interpretation of data, DND extraction, PCR, drafted, revised, and finalized the manuscript for submission. All authors have read and approved the final manuscript.
